# Progesterone inhibits endometrial cancer growth by inhibiting glutamine metabolism through ASCT2

**DOI:** 10.1042/BSR20232035

**Published:** 2024-03-12

**Authors:** Jinqiu Guo, Jianhui Fan, Yaru Zhang, Mengyue Li, Zeen Jin, Yuhong Shang, Hongshuo Zhang, Ying Kong

**Affiliations:** 1 Department of Biochemistry and Molecular Biology, College of Basic Medical Sciences, Dalian Medical University, Dalian, China; 2 Department of Gynecology, First Affiliated Hospital of Dalian Medical University, Dalian, China; 3 Advanced Institute for Medical Sciences, Dalian Medical University, Dalian, China

**Keywords:** ASCT2, Endometrial carcinoma, Glutamine metabolism, Progesterone

## Abstract

Endometrial carcinoma (EC) is a common malignancy that originates from the endometrium and grows in the female reproductive system. Surgeries, as current treatments for cancer, however, cannot meet the fertility needs of young women patients. Thus, progesterone (P4) therapy is indispensable due to its effective temporary preservation of female fertility. Many cancer cells are often accompanied by changes in metabolic phenotypes, and abnormally dependent on the amino acid glutamine. However, whether P4 exerts an effect on EC via glutamine metabolism is unknown. In the present study, we found that P4 could inhibit glutamine metabolism in EC cells and down-regulate the expression of the glutamine transporter ASCT2. This regulation of ASCT2 affects the uptake of glutamine. Furthermore, the *in vivo* xenograft studies showed that P4 inhibited tumor growth and the expression of key enzymes involved in glutamine metabolism. Our study demonstrated that the direct regulation of glutamine metabolism by P4 and its anticancer effect was mediated through the inhibition of ASCT2. These results provide a mechanism underlying the effects of P4 therapy on EC from the perspective of glutamine metabolism.

## Introduction

Endometrial carcinoma (EC) is a common malignancy of the female reproductive system derived from the endometrial epithelium. EC has the second highest incidence rate for malignant tumors in the female reproductive system in China, which ranks second only to cervical cancer [1]. It has the fourth-highest incidence rate in the United States [2]. The incidence of EC has increased in all countries in the world in recent years, and the age of onset has decreased [3]. The incidence of EC is closely related to metabolic syndrome including obesity, hyperglycemia, dyslipidemia, and hypertension [4]. Studies have found that obesity may increase the risk of EC through estrogen, insulin-like growth factor (IGF), inflammatory factors, and adipokines [5,6]. High triglyceride levels due to dyslipidemia may increase the risk of EC by increasing cellular estrogen levels or promoting epithelial–mesenchymal transition [7,8]. Diabetes mellitus (especially Type 2 diabetes) exhibits insulin resistance and hyperinsulinemia, which promotes EC through the IGF pathway [9]. This suggests a strong link between metabolism and EC. Metabolic reprogramming often occurs in tumor cells, with major changes occurring in several energy metabolic pathways [10,11], including glucose transport, oxidative phosphorylation of the mitochondrial respiratory chain, the (tricarboxylic acid) TCA cycle, and glutamine decomposition. Recent studies have shown that cancer cells have the highest glutamine intake in the tumor microenvironment [12], and glutamine metabolism can stimulate cell viability in endometrial carcinoma [13].

Glutamine (Gln) is one of the most abundant amino acids in humans and plays an important role in many metabolic processes [14]. Gln is metabolized into α-ketoglutaric acid and enters the Krebs cycle to provide energy for cells and precursors for macromolecule synthesis. Gln can also be broken down to glutamate, directly converted or reconverted to other amino acids, or be used as a nitrogen source for purine and pyrimidine synthesis and the hexosamine biosynthesis pathway [15]. The increased glutamine demand by tumor cells enhances glutamine metabolism to compensate for the intermediates required by the tricarboxylic acid cycle, which satisfies the energy requirements for biomolecular synthesis and redox homeostasis of tumor cells and promotes glutamine-dependent tumor cell growth [16]. However, the molecular mechanisms regulating glutamine metabolism in EC are poorly understood.

Glutamine carrier 2 (ASCT2), also known as solute carrier family 1 member 5 (SLC1A5), is a Na+-dependent neutral amino acid transporter protein that mediates the intracellular influx and efflux of glutamine. ASCT2 is important for Gln intake in EC cells [17] and a key transport protein for Gln metabolism in EC [18]. Furthermore, ASCT2 is up-regulated in various cancers, including tongue [19], lung [20,21], breast [18], pancreatic [22], gastric [23,24], colorectal [25], prostate [26], and renal cell cancer [27,28]. ASCT2 is also up-regulated in EC [17]; this suggests an important role for glutamine metabolism in the development of EC.

P4 therapy is a feasible treatment for EC. Indeed, a 2015 study showed that 39 of 53 patients had a complete response after six months of oral P4, and 17 of 33 patients could successfully conceive [29]. As a result of its effective temporary preservation of female fertility, P4 therapy has become an indispensable treatment option for young patients in the past few years [29]. However, it is unclear whether P4 affects EC by modulating Gln metabolism. The present study aimed to investigate the relationship between P4 and Gln metabolism and its role in the progression of EC in vivo and in vitro and to understand the mechanism of P4 in the treatment of EC.

## Materials and methods

### Clinical samples

Endometrial tissues (*n* = 7 normal and 13 tumors) were obtained from the First Affiliated Hospital of Dalian Medical University. The pathology of the samples was verified by pathologists through hematoxylin-eosin (HE) staining. All patients supplied written informed consent, and the study was approved by the Ethics Committee (PJ-KS-KY-2022-436) from the First Affiliated Hospital of Dalian Medical University.

### Cell culture

The EC cell lines Ishikawa and RL95-2 were purchased from the Cell Bank of the Chinese Academy of Sciences (Shanghai, China). The Ishikawa cells were grown in RPMI 1640 medium supplemented with 10% fetal bovine serum (FBS). RL95-2 cells were grown in DMEM/F-12 medium supplemented with 0.005 mg/ml insulin and 10% FBS.

### RNA interference and ASCT2 overexpression

Cells were grown in 35 mm culture dishes and cultured to 70% density. For down-regulated ASCT2, 100 mM ASCT2-targeting siRNA or negative controls (scrambled sequence) were transfected with 5 μl Lipofectamine 2000 (Invitrogen, U.S.A.), the interference sequence is: Negative control sense: 5′-UUCUCCGAACGUGUCACGUTT-3′, Negative control antisense: 5′-ACGUGACACGUUCGGAGAATT-3′, ASCT2-homo sense: 5′-GCCUUGGCAAGUACAUUCUTT-3′, ASCT2-homo antisense: 5′-AGAAUGUACUUGCCAAGGCTT-3′. For overexpression of ASCT2, 5ug pcDNA3.1(+)-Flag-ASCT2 overexpression plasmid or empty plasmid (constructed by PPL Co. Ltd, China) was transfected with 5 μl Lipofectamine 2000 (Invitrogen, U.S.A.).

### Cell proliferation and migration assays

Cell proliferation and viability were examined using the Cell Counting Kit-8 (CCK-8) assay (Dojindo, Japan). After treatment with different P4 concentrations (0, 1, 10, and 20 µM), the cells were tested according to the manufacturer’s instructions. For the cell scratch assay, Ishikawa and RL95-2 cells were counted and seeded in 6-well plates. The cell monolayer was scratched with a sterile 100-μl pipette tip after serum starvation for 6 h. After treatment with P4 for 48 h, the wound gap was photographed with an inverted microscope. For the migration assay, Ishikawa and RL95-2 cells (5 × 10^4^) were seeded into the upper transwell chambers. The upper and bottom chambers contained the same concentration of P4. After 48 h, the infiltrated cells were stained with 0.1% Crystal Violet and photographed under a microscope.

### Glutamine uptake assay and ATP assay

The glutamine content in the cell supernatant was detected using a Glutamine Content Kit (Jianglai Biology, China) according to the manufacturer’s instructions. The net glutamine intake was determined by subtracting the glutamine content of the supernatant from the glutamine content of the original medium. ATP level was assayed as previously reported [30], and the analysis of ATP content after P4 and/or CB-839 treatment of cells was performed according to the kit instructions (Solarbio, Beijing, China).

### Immunohistochemistry (IHC)

Paraffin sections of endometrial tissue were dewaxed in xylene and hydrated with decreasing ethanol concentrations. Antigen recovery was performed in citric acid buffer (pH 6.0). The sections were blocked with FBS for 20 min and incubated overnight at 4°C with an ASCT2-specific (Proteintech, China; 20350-1-AP, 1,000 μg/ml) or GLS-specific (Proteintech, China; 23549-1-AP, 1,000 μg/ml) antibody at a dilution of 1:200. Immunodetection was performed using the Diaminobenzidine-HRP Reaction System (ZSGB-Bio Co., China).

### Immunofluorescence (IF)

Ishikawa and RL95-2 cells were treated with P4 for 48 h and transferred to slides, 6 h later cells were fixed with 4% paraformaldehyde and blocked with goat serum followed by overnight incubation with ASCT2 antibody (1:200) at 4°C. CoraLite594-conjugated secondary antibody (1:25) was incubated under dark conditions at 37°C for 1 h, followed by staining with DAPI for 5 min at room temperature, and the images were visualized using a fluorescence microscope.

### Real-time polymerase chain reaction (PCR) analysis

Total RNA was extracted from the cells using a Trizol reagent (Thermo Fisher Scientific, U.S.A.). Reverse transcription was performed with the PrimeScript RT Reagent Kit (TaKaRa Bio, Japan). Real-time PCR was performed using the SYBR Premix Ex Taq II Kit (TaKaRa Bio, Japan) with the CFX96 Real-Time PCR Detection System (Bio-Rad Laboratories, U.S.A.). The expression levels of the target genes were normalized to the reference gene (β-actin) using the 2^−ΔΔCT^ method. The primers are listed in Table 1.

**Table 1:** Primers

Name	Sequences
Homo-ASCT2 forward primer:	5′-CCGCCTTGGCAAGTACATTCT-3′
Homo-ASCT2 reverse primer:	5′-GGCAGGATGAAACGGCTGA-3′
Homo-GLS forward primer:	5′-AGGGTCTGTTACCTAGCTTGG-3′
Homo-GLS reverse primer:	5′-ACGTTCGCAATCCTGTAGATTT-3′
Homo-β-actin forward primer:	5′-TGTTTGAGACCTTCAACACC-3′
Homo-β-actin reverse primer:	5′-ACGCAGGATGGCATGG-3′

### Western blotting

The proteins were extracted with lysis buffer (KeyGen BioTECH, China). The proteins (30 μg) were separated by 10% sodium dodecyl sulfate-polyacrylamide gel electrophoresis and transferred to a nitrocellulose membrane (Millipore, U.S.A.). The membranes were blocked with 5% skim milk and incubated with an antibody to ASCT2 (Proteintech, China; 20350-1-AP, 1:2000), GLS (Proteintech, China; 23549-1-AP, 1:2000), or β-actin (Proteintech, China; 20536-1-AP, 1:1,000) overnight at 4°C. The signal was detected with an HRP-conjugated secondary antibody (Proteintech, China, 1:3,000) for 1 h at room temperature and visualized using an ECL kit (Thermo Fisher Scientific, U.S.A.).

### EC xenografts

Eight-week-old female BALB/c nude mice were provided by Beijing VTC Lihua Experimental Animal Technology Co, Ltd. and were housed at the Specific Pathogen Free (SPF) Experimental Animal Center of Dalian Medical University. The animal experiments were performed at the SPF Experimental Animal Center of Dalian Medical University and were reviewed and approved by the Animal Ethics Committee of Dalian Medical University (project approval NO.: AEE21072). After disinfecting the left anterior axilla with iodophor, the nude mouse was injected with a mixture containing 100 µl of 5 × 10^7^ Ishikawa cells and Matrigel (BD Biosciences, U.S.A.). The tumor-bearing mice were divided into control and P4 treatment groups (*n*=7). The control (30% cyclodextrin) and P4 (0.63 mg/g in 30% hydroxypropyl-β-cyclodextrin) were subcutaneously administered in the posterior neck of the mice in 100 μl daily at the same time of day. Mice were weighed twice per week, and the length and width of the tumors were measured with a vernier caliper. The mice were euthanized (sodium pentobarbital: 150 mg/kg intraperitoneal) after 28 days, and the tumors were resected and weighed, measured in length and width, and immersed in formalin or preserved in liquid nitrogen.

### Statistical analysis

Data are represented as the mean ± standard deviation for a minimum of three independent experiments. Groups were compared by the Student’s *t*-test and one-way ANOVA using GraphPad Prism 8.0 (GraphPad Software). *P*<0.05 was considered statistically significant.

## Results

### P4 inhibits glutamine metabolism in EC cells

**Figure 1:**
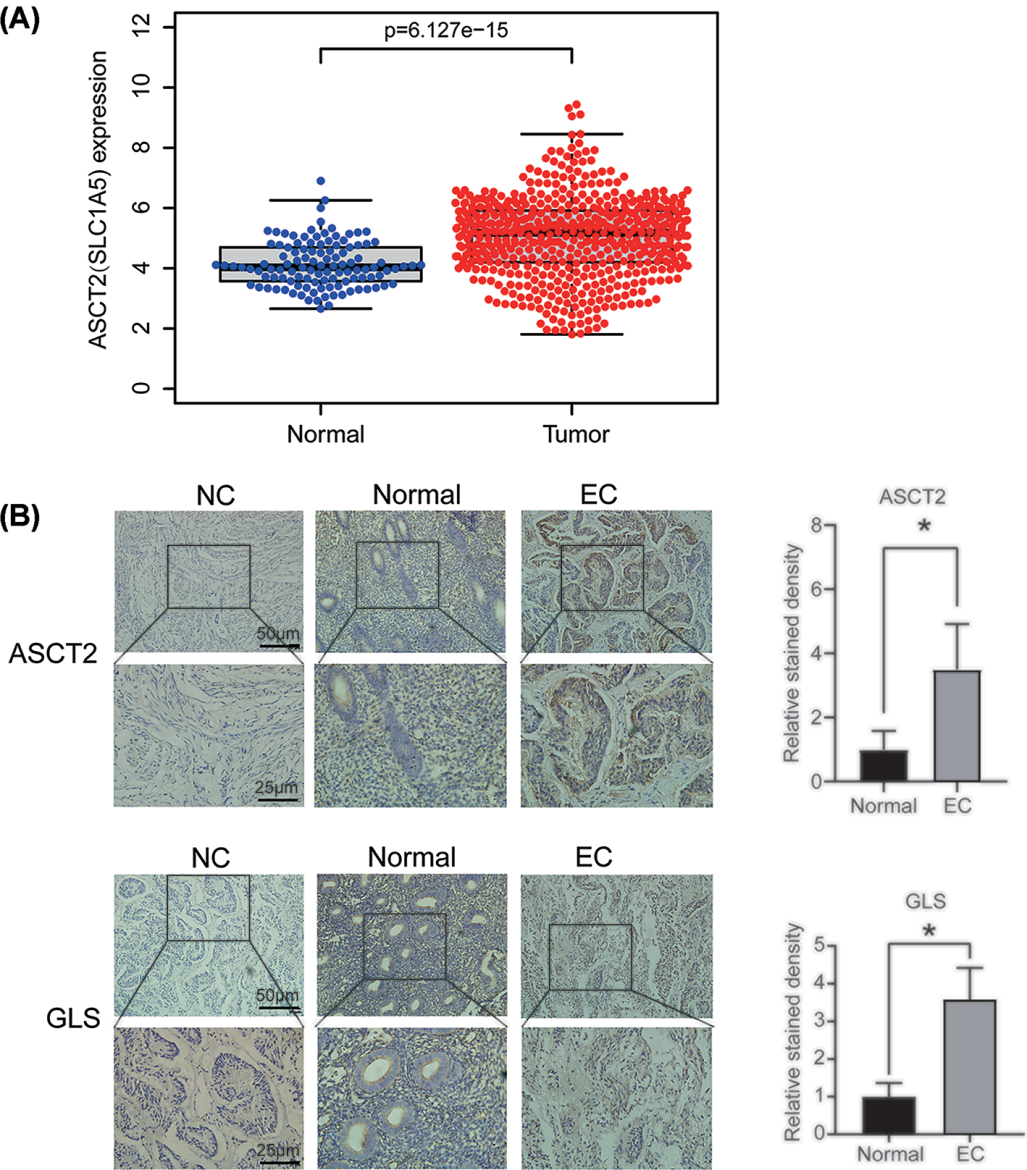
Expression of key enzymes of glutamine metabolism in EC tissues (**A**) TCGA database analysis of ASCT2 expression in normal endometrium (*n*=113) and EC (*n*=548) (*P* = 6.127 × 10^-15^). (**B**) Immunohistochemical (IHC) staining of ASCT2 and GLS in human EC tissues and normal endometrial tissues (*n*=7) and EC tumors (*n*=13). Scale bars: 50 and 25 µm; *P<0.05.

By TCGA database analysis, we found that ASCT2 expression was significantly higher in EC patients than in normal endometrial tissue (Figure 1A). Using IHC, we also detected the protein expression of ACST2 and glutaminase (GLS), the key enzyme for converting glutamine into glutamate, in EC and normal endometrial tissue. The results showed that ASCT2 and GLS levels were higher in EC than in normal tissue, and the two proteins were mainly located in the glandular epithelium (Figure 1B), suggesting that the uptake and metabolism of glutamine were enhanced in EC cells.

**Figure 2:**
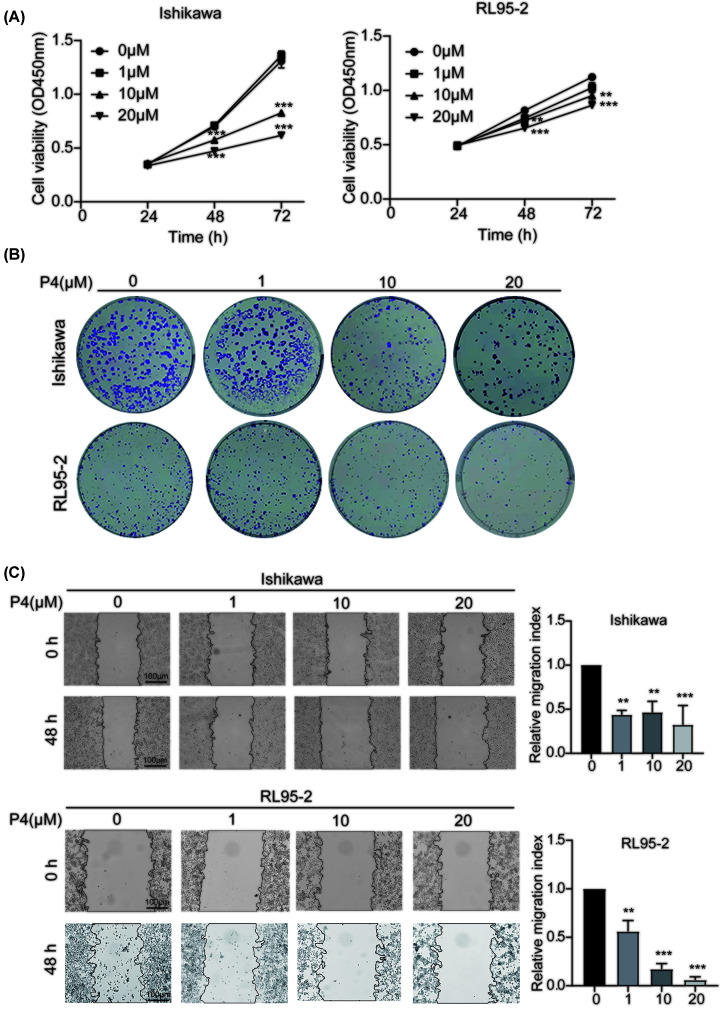
Effects of P4 on the proliferation and migration of EC cells (**A**) The effects of different concentrations (0, 1, 10, and 20 µM) of P4 on the proliferation of Ishikawa and RL95-2 EC cells were analyzed using the CCK-8 assay (*n*=3). (**B**) The effects of different concentrations (0, 1, 10, and 20 µM) of P4 on the Ishikawa and RL95-2 EC cells were analyzed using the colony formation assay (*n*=3). (**C**) Cell scratch assay analyzing the effects of different concentrations of progestins on the migration of Ishikawa and RL95-2 EC cells. Scale bars: 100 µm (*n*=3); **P<0.01, ***P<0.001.

To determine the effects of P4 on EC cells, Ishikawa and RL95-2 cells were treated with different concentrations of P4 (0, 1, 10, and 20 µM) for 48 h. The proliferation of EC cells was inhibited by as little as 10 µM P4 (Figure 2A). This finding was confirmed by the colony formation assay (Figure 2B). Furthermore, the migration of Ishikawa and RL95-2 cells was significantly inhibited in the scratch assays by 10 and 20 µM P4 (Figure 2C).

**Figure 3:**
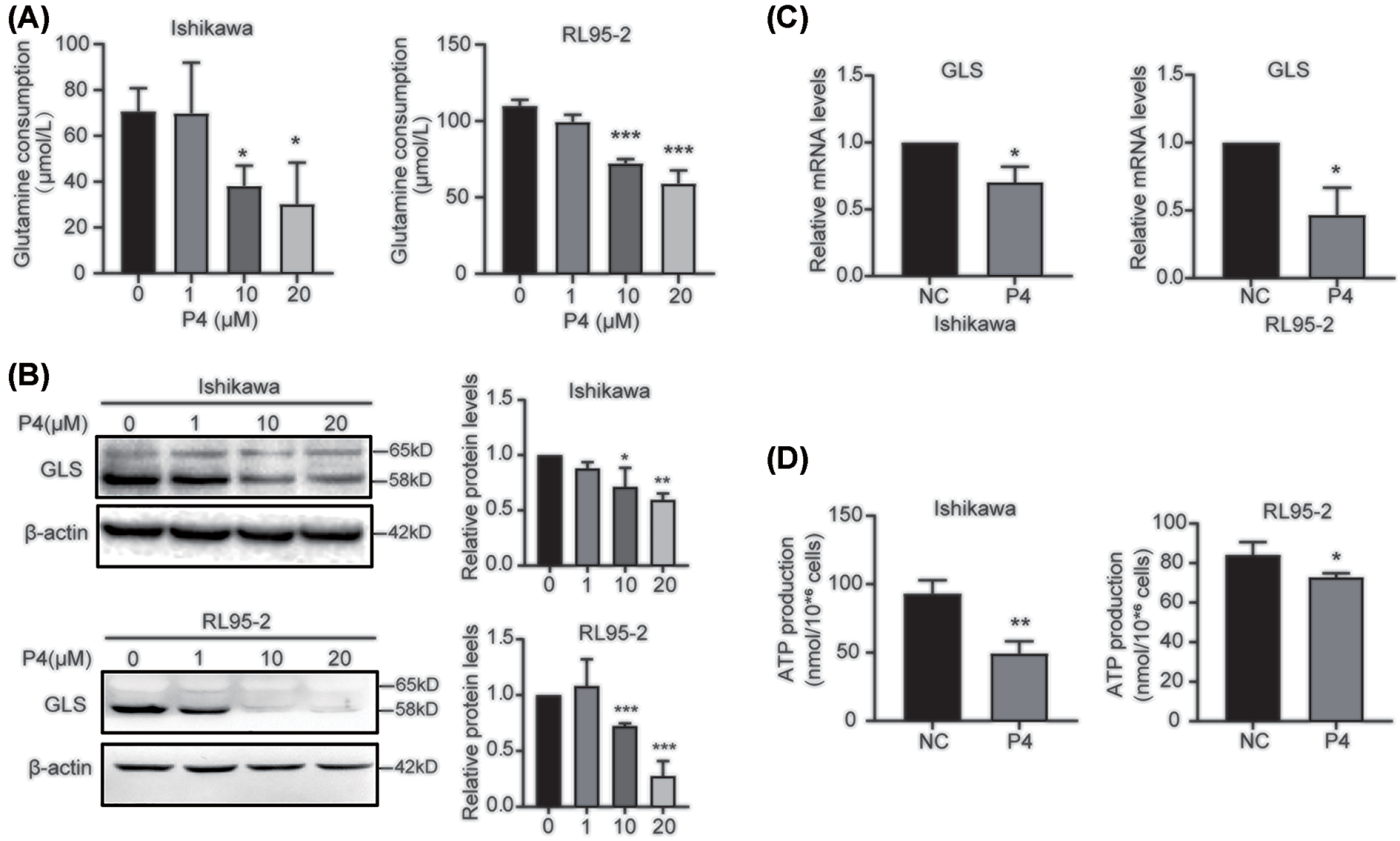
Effects of P4 on glutamine metabolism in EC cells (**A**) The effects of P4 (0, 1, 10, and 20 µM) on glutamine consumption in the supernatant from EC cells was determined by ELISA (*n*=3). (**B**) Western blot analysis of GLS protein expression in Ishikawa and RL95-2 EC cells after P4 treatment (*n*=3). (**C**) qPCR analysis of GLS mRNA expression in P4-treated Ishikawa and RL95-2 EC cells using β-actin as the reference gene (*n*=3). (**D**) The ATP level was detected by ATP assay kits after treatment with P4 (10 μM, *n*=3); *P<0.05, **P<0.001, ***P<0.001.

To investigate the effect of P4 on glutamine metabolism in EC cells, we examined the glutamine content in culture supernatants from Ishikawa and RL95-2 cells treated with different concentrations of P4 (0, 1, 10, and 20 µM) and found that high concentrations of P4 (10–20 µM) significantly reduced glutamine consumption (Figure 3A), suggesting decreased cellular uptake of glutamine. In addition, high P4 concentrations (10 µM) reduced GLS protein and mRNA expression (Figure 3B,C), and inhibited ATP production (Figure 3D). These results indicate that P4 could reduce the proliferation and migration of EC cells and inhibit glutamine metabolism.

### P4 inhibits ASCT2 expression in EC cells

**Figure 4:**
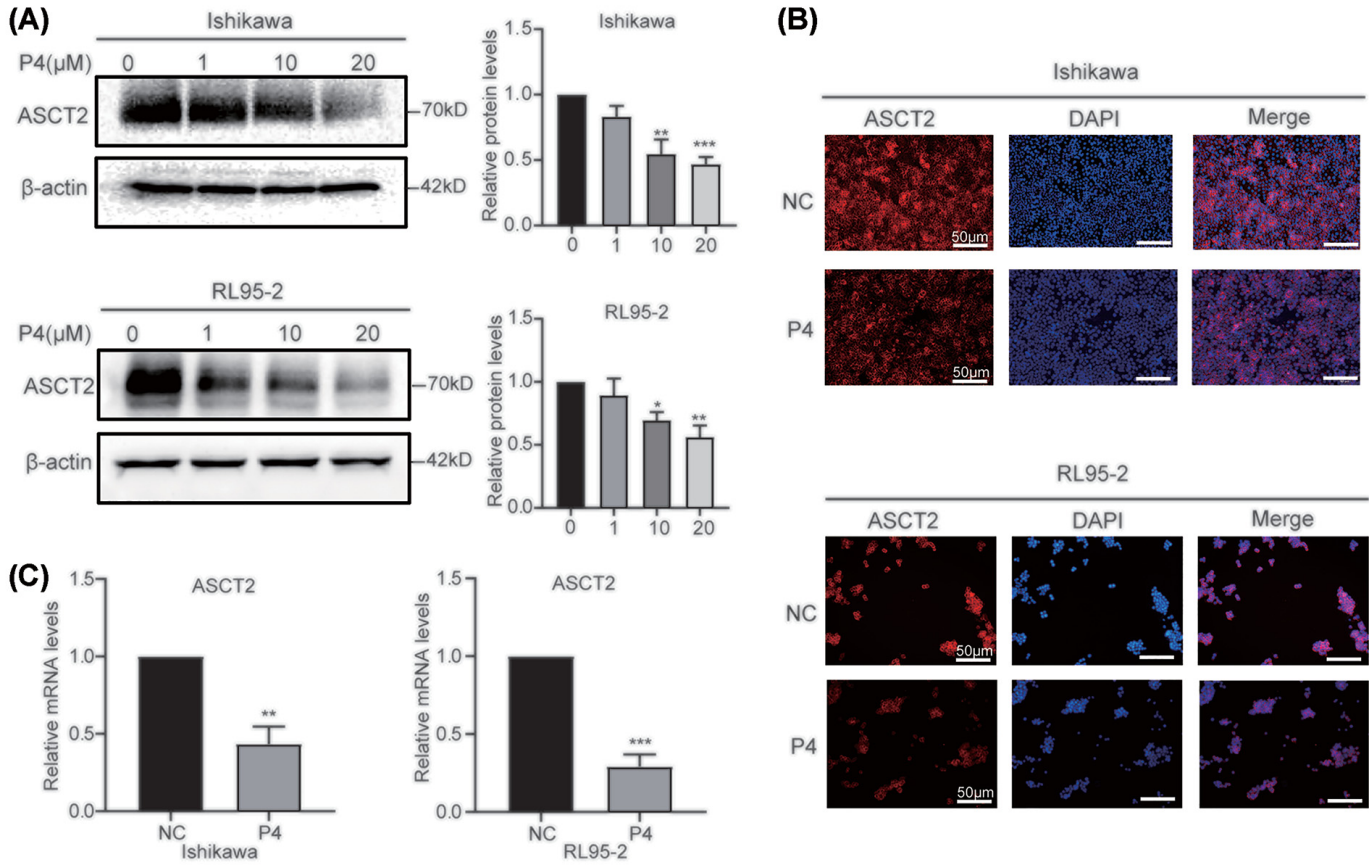
Effects of P4 on the expression of ASCT2 (**A**) Western blot analysis of ASCT2 protein expression in Ishikawa and RL95-2 EC cells (*n*=3). (**B**) ASCT2 immunofluorescence in Ishikawa and RL95-2 EC cells following treatment with P4 (*n*=3, scale bars = 50 μm). (**C**) qPCR analysis of ASCT2 mRNA expression in Ishikawa and RL95-2 EC cells treated with P4 using β-actin as the reference gene (*n*=3); *P<0.05, **P<0.001, ***P<0.001.

To elucidate the effect of P4 on ACST2, we found that high concentrations of P4 (10–20 µM) significantly inhibited ASCT2 protein expression (Figure 4A). Moreover, 10 µM P4 decreased ASCT2 protein expression detected by immunofluorescence (Figure 4B) and reduced ACST2 mRNA levels measured by real-time PCR (Figure 4C).

### Down-regulation of ACST2 inhibits glutamine metabolism and reduces the proliferation and migration of EC cells

**Figure 5:**
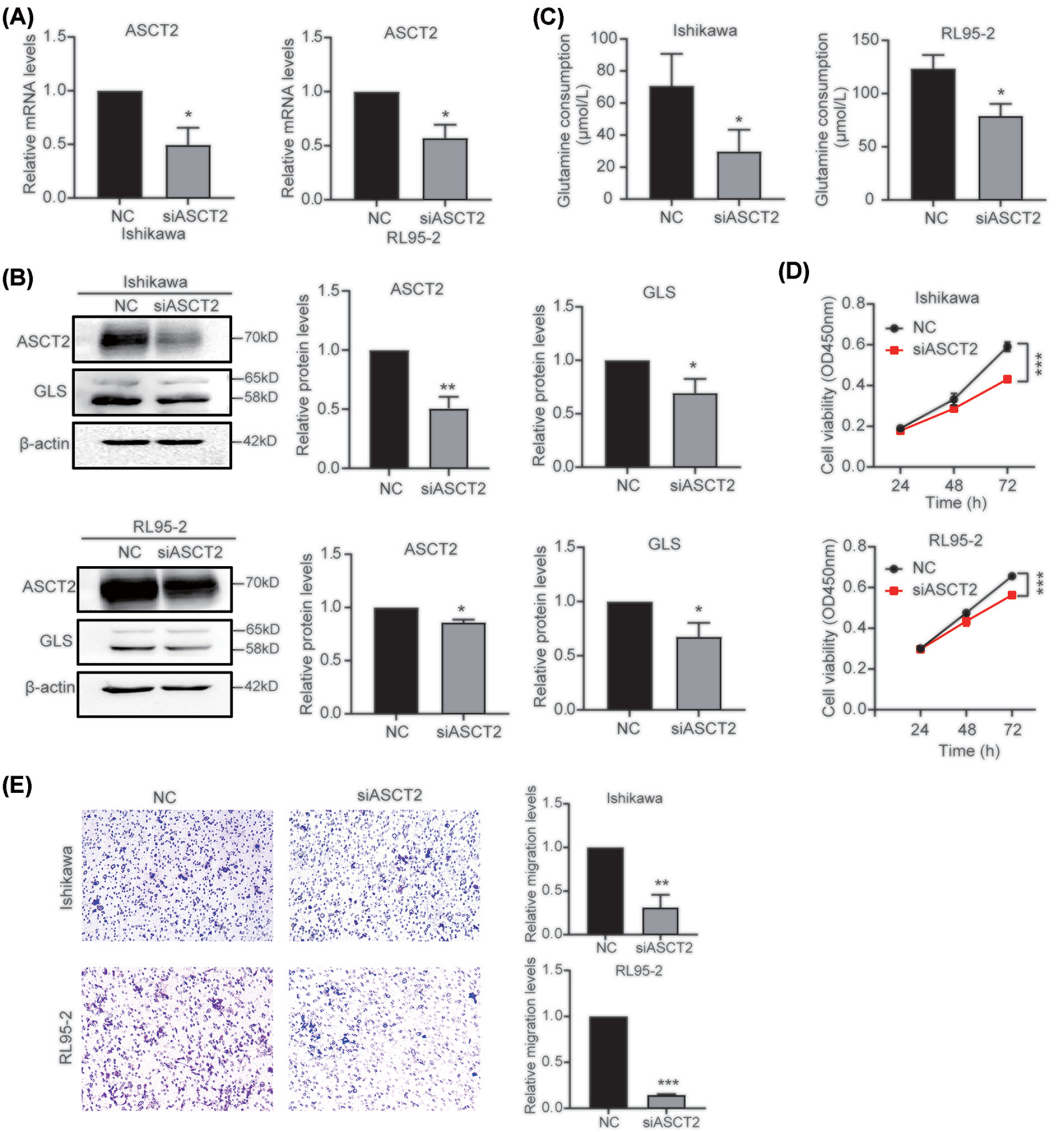
Effects of down-regulating ASCT2 on glutamine metabolism and the proliferation and migration of EC cells (**A**) qPCR analysis of the efficiency of siRNA-mediated ASCT2 down-regulation using β-actin as the reference gene (*n*=3). (**B**) Western blot analysis of ASCT2 protein expression in EC cells transfected with siASCT2 (*n*=3). (**C**) Effects of ASCT2 down-regulation on glutamine consumption in EC cell supernatants (*n*=3). (**D**) The effects of ASCT2 down-regulation on the proliferation of Ishikawa and RL95-2 EC cells were analyzed using the CCK-8 assay (*n*=3). (**E**) The effects of ASCT2 down-regulation on the migration of Ishikawa and RL95-2 EC cells were analyzed using the transwell assay (*n*=3); *P<0.05, **P<0.001, ***P<0.001.

To verify the role of ACST2, we down-regulated ACST2 expression using small interfering RNA (siRNA) (Figure 5A,B) and found that ASCT2 down-regulation resulted in a significant decrease in glutamine consumption in Ishikawa and RL95-2 cells (Figure 5C). This down-regulation also decreased GLS protein expression (Figure 5B), suggesting that glutamine metabolism may be inhibited. Moreover, the proliferation of EC cells and their migration were reduced by ASCT2 siRNA (Figure 5D,E), indicating that regulating ACST2 levels could alter EC cell proliferation and migration.

### P4 reduces EC cell glutamine metabolism, proliferation, and migration by inhibiting ASCT2

**Figure 6:**
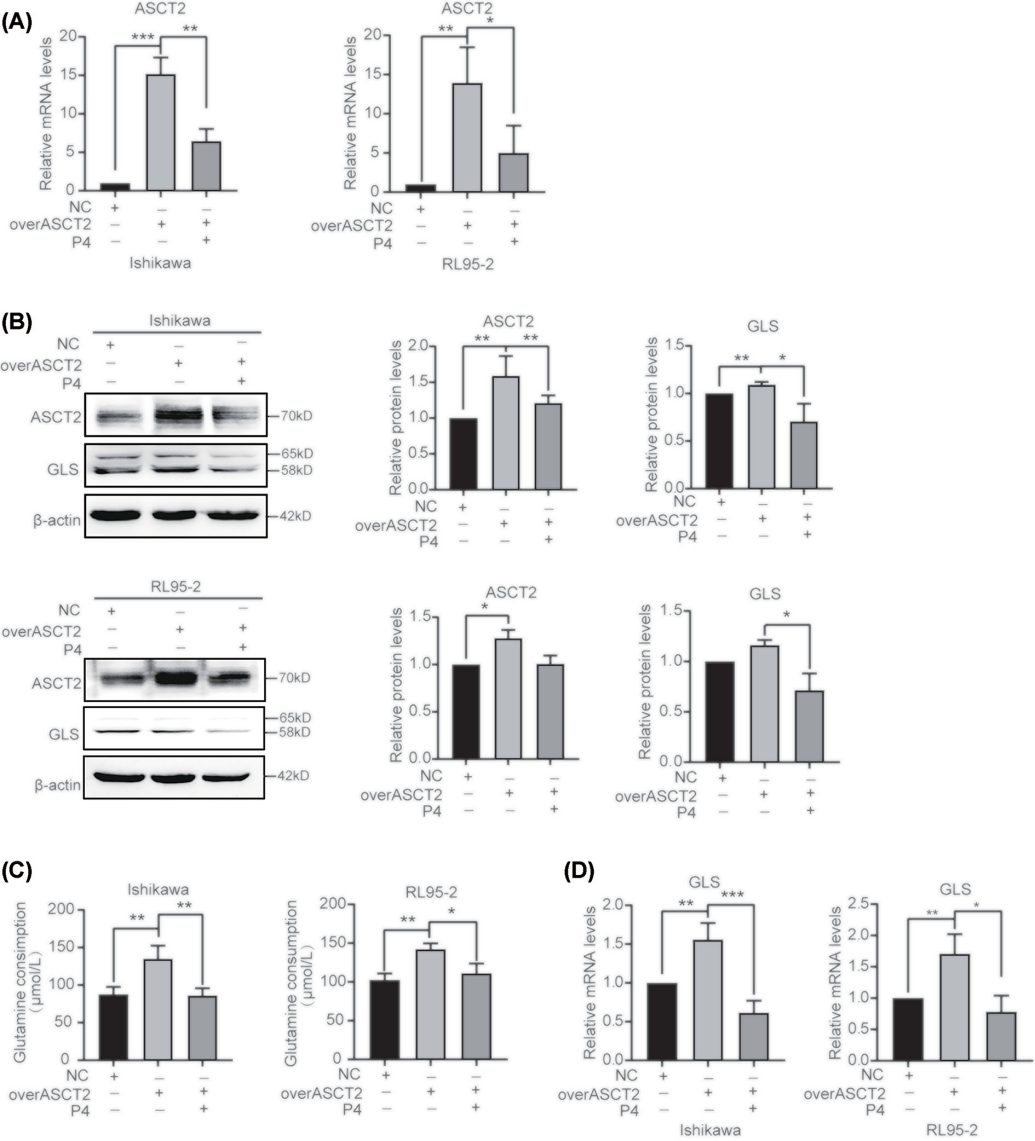
P4 inhibits glutamine metabolism via ASCT2 (**A**) qPCR analysis of ASCT2 mRNA expression in EC cells following ASCT2 overexpression and P4 treatment (*n*=3). β-Actin was used as the reference gene. (**B**) Western blot analysis of ASCT2 and GLS protein expression in EC cells following ASCT2 overexpression and P4 treatment (*n*=3). (**C**) Effects of ASCT2 up-regulation and P4 treatment on glutamine consumption in EC cell supernatants (*n*=3). (**D**) qPCR analysis of GLS mRNA expression in EC cells following ASCT2 overexpression and P4 treatment (*n*=3). β-Actin was used as the reference gene; *P<0.05, **P<0.001, ***P<0.001.

**Figure 7:**
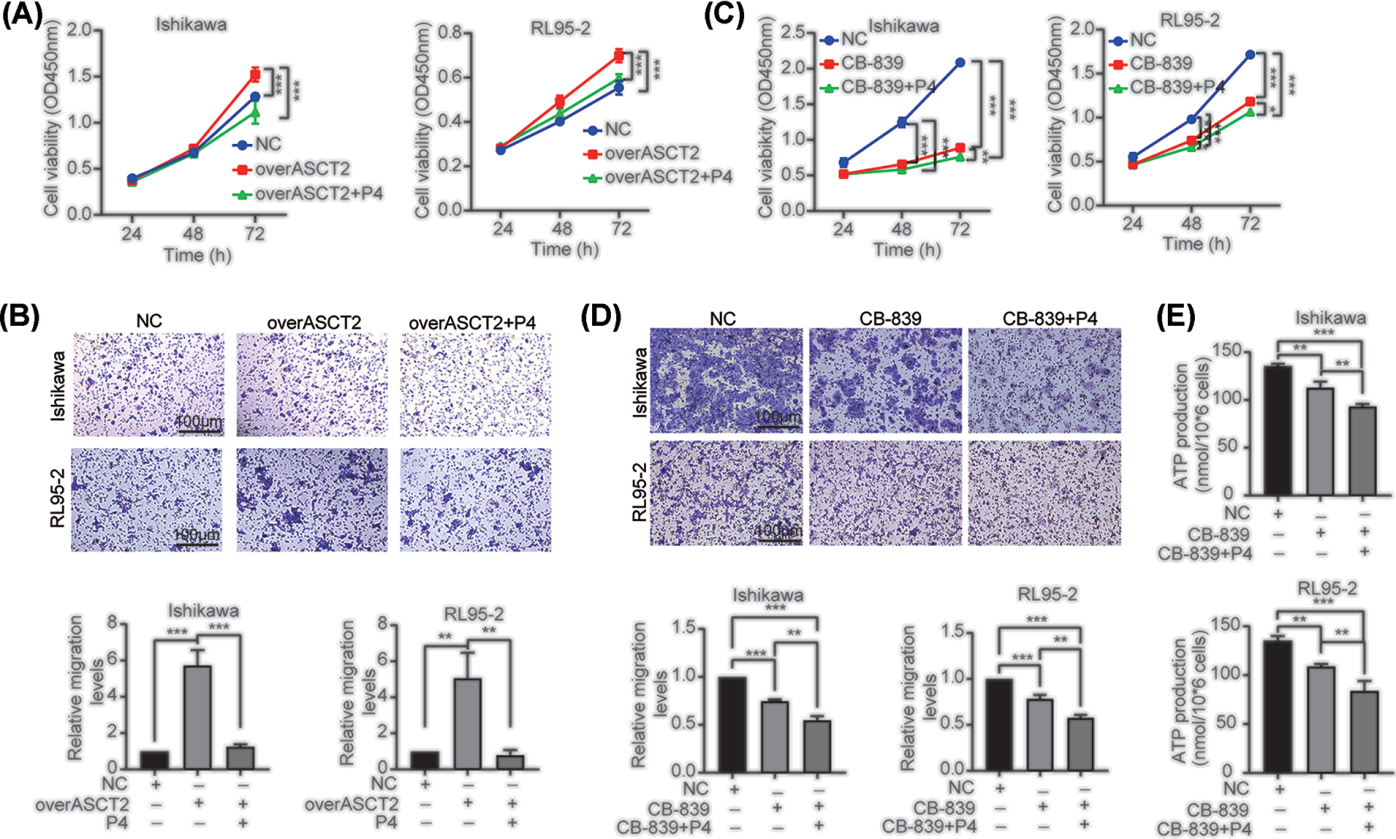
P4 inhibits EC cell proliferation and migration via ASCT2 (**A**) Effects of ASCT2 up-regulation and P4 treatment on the proliferation of Ishikawa and RL95-2 EC cells were analyzed using the CCK-8 assay (*n*=3). (**B**) The effects of ASCT2 up-regulation and P4 treatment on the migration of Ishikawa and RL95-2 EC cells were analyzed by the Transwell assay (scale bars = 50 µm, *n*=3). (**C**) Effects of CB-839 (1μM) and P4 treatment on the proliferation of Ishikawa and RL95-2 EC cells were analyzed using the CCK-8 assay (*n*=3). (**D**) The effects of CB-839 and P4 treatment on the migration of Ishikawa and RL95-2 EC cells were analyzed by the Transwell assay (scale bars = 50 µm, *n*=3). (**E**) The ATP level was detected by ATP assay kits after treatment with CB-839 and P4 (10 μM, *n*=3); *P<0.05, **P<0.001, ***P<0.001.

To verify whether P4 regulates glutamine metabolism through ASCT2, we overexpressed ASCT2 in EC cells, the mRNA and protein levels confirmed the up-regulation of ASCT2 (Figure 6A,B). We then found that the up-regulation of ASCT2 induced the consumption of glutamine (Figure 6C), which was inhibited by P4 (Figure 6A–C). Moreover, P4 inhibited ASCT2 and GLS mRNA and protein expression (Figure 6A,B,D). P4 also inhibited the increased proliferation and migration of EC cells caused by ASCT2 overexpression (Figure 7A,B). Subsequently, we treated the cells with CB-839 (1 μM), an inhibitor of GLS, and found that inhibition of GLS reduced proliferation, migration, and ATP production levels in endometrial cancer cells, while concurrent treatment with P4 further reduced these levels (Figure 7C–E). These results suggest that P4 could reduce glutamine metabolism in EC cells by inhibiting ASCT2, thereby abrogating proliferation and migration and reducing the malignant behavior of EC cells.

### P4 inhibits tumor growth and the expression of key enzymes of glutamine metabolism in EC xenografts

**Figure 8:**
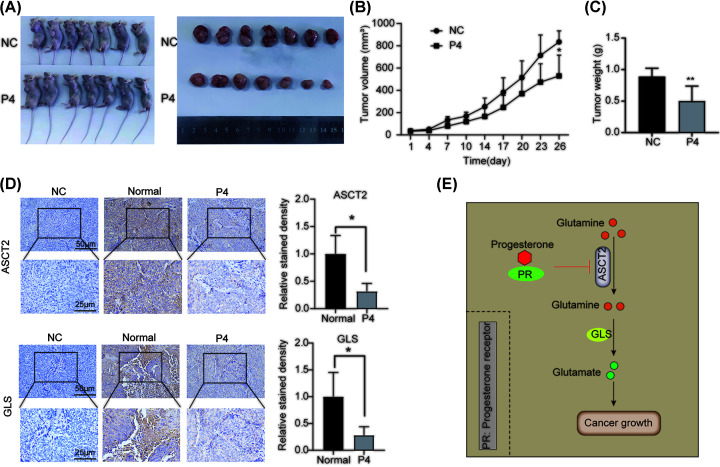
P4 inhibits the expression of key enzymes in glutamine metabolism and tumor growth *in vivo* (**A**) Photographs of tumors in xenograft mice. (**B**) Tumor volumes for the P4 and control treatment groups (*n*=7). (**C**) Tumor weights for the P4 and control treatment groups (*n*=7). (**D**) Immunohistochemical analysis of ASCT2 and GLS expression in tumor tissues from the P4 and control treatment groups (*n*=6). Scale bars: 50 and 25 µm. (**E**) Schematic diagram of P4 regulating glutamine metabolism via ASCT 2; *P<0.05, **P<0.001.

To determine whether P4 could inhibit glutamine metabolism *in vivo*, Ishikawa tumor-bearing nude mice were treated with 0.63 mg/g in 30% hydroxypropyl-β-cyclodextrin (P4 group) or 30% hydroxypropyl β-cyclodextrin (control group) daily for 28 days. The tumor volumes and weights were significantly lower in the P4 group than in the control group (Figure 8A–C). IHC analysis demonstrated that the expression levels of ASCT2 and GLS, a key enzyme in glutamine metabolism, were lower in the P4 group than in the controls (Figure 8D). These results suggest that P4 inhibited glutamine metabolism *in vivo* by decreasing ASCT2 expression, thereby suppressing the proliferation, migration, and other malignant behaviors of EC cells.

## Discussion

EC is one of the most common invasive gynecological cancers worldwide; however, a proportion of EC patients are younger than 40 years of age [31]. As women increasingly delay childbearing, 70% of EC patients under the age of 40 have fertility needs [32]. P4 therapy can meet the requirements for treatment and fertility for these EC patients [33,34]. In 1961, Kelley and Baker [35] observed that P4 was beneficial for patients with advanced EC, which was confirmed by later research [36–38]. Although P4 therapy has become a feasible therapy for EC, the specific mechanism underlying the effect of P4 on EC has yet to be deeply investigated [39,40].

EC has traditionally been divided into two groups [41]: type I and type II. Type I EC accounts for 80–90% of EC cases with a better prognosis and a low recurrence rate (20%) compared with type II EC [42]. The main characteristics of type I EC patients are hyperfunction of the thalamus, ovary, and other glands and metabolic disorders. Typical manifestations include primary or secondary infertility, endometrial proliferation, ovarian interstitial hyperplasia, and anovulatory uterine bleeding. Type II EC lacks the symptoms of increased estrogen and does not cause menstrual and metabolic disorders. The prognosis for patients with type II EC is poor (58.8% 5-year survival rate) [43]. Type I EC is also known as estrogen-dependent EC, and the main risk factor is excessive estrogen exposure. P4 antagonizes estrogen, and the side effects of P4 are mild. Thus, P4 therapy has been and will continue to be a viable treatment for type I EC.

Heterogeneous metabolism is common in cancer, and cancer cells often use the precursors of TCA cycle intermediates to synthesize proteins, lipids, and nucleic acids; reduced TCA cycle intermediate levels make it difficult to maintain mitochondrial activity [44]. Tumor cells must compensate for this deficiency caused by their metabolic metastasis [45]. Glutamine is metabolized into α-ketoglutaric acid, which enters the TCA cycle to provide energy for cells. It also serves as a nitrogen and carbon source for cells involved in purine and pyrimidine synthesis and the hexosamine biosynthesis pathway. The increased glutamine demand of tumor cells can provide intermediates for the TCA cycle to complement energy metabolism, biomolecular synthesis, and redox homeostasis. Studies have shown that glutamine metabolism can stimulate the cell viability of EC cells [13]. Glutamine enters the cell by active transport via a glutamine transporter, where it is hydrolyzed to glutamate and ammonia by GLS. Glutamate can be catalyzed into α-ketoglutarate by glutamate dehydrogenase (GDH) and enter the TCA cycle [46]. Notably, ASCT2 is overexpressed in a variety of cancer cells and is thought to be a major transporter of glutamine in these cancer cells [47]. Therefore, inhibition of ASCT2 as an antitumor therapy is very promising. In 2017, Marshall et al. found that ASCT2 was up-regulated in different subtypes of endometrial cancer, and that inhibiting glutamine uptake or down-regulating ASCT2 in vitro can reduce the growth of endometrial cancer cells [17]. This is a strong indication of the important role of ASCT2-mediated glutamine metabolism in endometrial cancer cells, which is validated by our study and the findings that glutamine metabolism can be affected by P4 signaling, and that P4 inhibits glutamine uptake by suppressing ASCT2 expression.

In the present study, we found that ASCT2 was differentially expressed in EC versus normal endometrial tissue by bioinformatics analysis. We also observed elevated expression of ASCT2 and GLS in EC tumor samples, suggesting altered glutamine metabolism. Furthermore, we verified the inhibitory effect of P4 on EC and found that P4 inhibited glutamine metabolism and ASCT2 expression. The regulation of ASCT2 levels (e.g., transfection with ASCT2 siRNA or ASCT2-overexpressing plasmid) could affect the level of glutamine metabolism, and P4 could inhibit the up-regulation of glutamine metabolism caused by ACST2 overexpression, thus inhibiting EC cell proliferation and migration. These results provide effective evidence for further study of EC pathogenesis and targeted intervention.

Our study shows that P4 can inhibit ACST2 expression and reduce glutamine uptake, thus inhibiting glutamine metabolism. It is unclear whether P4 directly targets ACST2. Studies have shown that c-Myc can directly up-regulate ASCT2 expression [48], and c-Myc is highly expressed in the serum and tumor tissue from EC patients [49]. P4 can decrease c-Myc protein levels in EC [50]. Therefore, P4 may regulate ASCT2 expression by decreasing c-Myc. In addition, P4 is an intracellular ligand that can directly bind to the promoter regions of some genes. Whether P4 is directly involved in the initiation of ASCT2 needs further investigation.

## Conclusions

Our study suggests that elevated glutamine metabolism provides energy and biosynthetic substrates for the proliferation and migration of EC cells. The regulation of ASCT2 affects the cellular uptake of glutamine, and P4 reduces glutamine uptake and decreases glutamine metabolism levels by inhibiting ASCT2 expression, thereby suppressing the malignant phenotype of EC. These data provide a reasonable explanation for the mechanism of P4 therapy for EC.

## Data Availability

All supporting data are included within the main article and its supplementary files.

## Abbreviations

ASCT2: glutamine carrier 2
EC: endometrial carcinoma
Gln: glutamine
GLS: glutaminase
IF: Immunofluorescence
IGF: insulin-like growth factor
IHC: immunohistochemistry
siRNA: small interfering RNA
TCA: tricarboxylic acid
